# Electric Volume Resistivity for Biopolyimide Using 4,4′-Diamino-α-truxillic acid and 1,2,3,4-Cyclobutanetetracarboxylic dianhydride

**DOI:** 10.3390/polym11101552

**Published:** 2019-09-24

**Authors:** Shunsuke Kato, Fitri Adila Amat Yusof, Toyohiro Harimoto, Kenji Takada, Tatsuo Kaneko, Mika Kawai, Tetsu Mitsumata

**Affiliations:** 1 Graduate School of Science and Technology, Niigata University, Niigata 950-2181, Japan; 2 Graduate School of Advanced Science and Technology, JAIST, Nomi 923-1292, Japan

**Keywords:** biopolymer, biopolyimide, polyimide, electric resistivity, electric resistivity

## Abstract

Biopolyimides poly(ATA-CBDA), made from of 4,4′-diamino-α-truxillic acid dimethyl ester (ATA) and 1,2,3,4-cyclobutanetetracarboxylic dianhydride (CBDA), is synthesized and measured its electric volume resistivity at various experimental conditions. The effects of film size, thickness, drying time, and the electric field strength on electric resistivity are investigated and compared with polyimide (Kapton). The electric resistivity for all polyimide and biopolyimide are distributed in the range of 10^15^–10^16^ Ωcm, which shows that biopolyimide has high electrical insulation as well as polyimide. The electric resistivity strongly depends on film thickness, which suggests that electric resistivity is a function of electric field strength. The critical electric field for polyimide and biopolyimide films are determined to be 5.8 × 10^7^ V/m and 3.2 × 10^7^ V/m, respectively. Humidity was found to strongly affect the electric resistivity; ~10^16^ Ωcm at 34% RH and ~10^13^ Ωcm at 60% RH for both polyimide and biopolyimide films.

## 1. Introduction

Soft polymer material is widely used as a material with electrical insulation properties. In general, the electric resistivity for polymers distributes in the range of 10^11^–10^16^ Ω cm. In particular, polyethylene (PE), cross-linked PE, and polyvinyl chloride (PVC) are widely used because they are good insulators for electrical wires and are able to endure high electric voltages. Over the last few years, the development of new polymers with high electrical insulation is very active due to requirements such as super high electric voltages, weight saving of electric devices, etc. Needless to say, polymers obtained from bioresources are far more preferable as materials that have high electrical insulation in the next generation. Since the last decade, biobased polymers are widely investigated and are produced in the materials currency. For example, poly(lactic acid)s (PLA), cellulose, and poly(buthylene succinate) (PBS) are famous biopolymers, and their composites, copolymers, and polymer blends have been extensively developed in recent years [1,2,3,4,5,6,7,8]. However, the defects in mechanical and thermal properties remain in some biobased polymers, which restrict their applications as engineering plastics.

Polyimide is a typical example of an engineering plastic that shows good thermal properties, with a glass transition temperature exceeding 300 °C and a high electrical insulation with a breakdown electric field strength of 400 kV/mm, and it is widely used in electronic industries, e.g., flexible printed electric circuits, flexible substrates for a chip of a semiconductor, and a coating material for electric motors [9,10,11,12]. Kaneko et al. extracted hydroxyacids from cinnamate, including p-hydroxycinnamic acid (4HCA), ferulic acid (3-methoxy-4-hydroxycinnamic acid; MHCA), caffeic acid (3,4-dihydroxycinnamic acid; DHCA), and 4-aminocinnamic acid (4ACA), and synthesized a copolymer of poly(4HCA-*co*-DHCA) with good mechanical properties [13]. In addition, it was also revealed that various biobased polyimides using 4,4′-diamino-α-truxillic acid dimethyl ester (ATA) demonstrate ultra-high thermal resistance with a 10% weight loss temperature of 425 °C [13]. It is worth mentioning that biopolyimide film is more transparent in comparison to polyimide, and is generally pale yellow to brown in color; this hinders its use as a flexible electrode for liquid-crystal displays or solar panels. The biopolyimide we use in this study have 4,4′-diamino-α-truxillic acid dimethyl ester (ATA) and 1,2,3,4-cyclobutanetetracarboxylic dianhydride (CBDA) as monomer units [14], and it demonstrates high Young’s modulus of 10 GPa and a high 10% weight loss temperature of 425 °C. In addition, the film of poly(ATA-CBDA) is transparent with a transmittance of 88% at 450 nm. Accordingly, the poly(ATA-CBDA) can be useful for a substrate with flexible electrodes if it has an advantage in electrical insulation properties. In this study, we measure the electric volume resistivity of a biopolyimide film, poly(ATA-CBDA), and discuss the various factors affecting the electrical insulation property of the films.

Another purpose of this study is to investigate the volume resistivity measurement for polymer films that are small in size. There are some international standards on the electric resistivity measurement for polymers, for example ASTM international (American Society for Testing and Materials) and IEC (International Electrotechnical Commission), etc. [15,16]. According to the standards, thick samples of several centimeters in size is needed. However, it is inconvenient for chemists or material scientists to prepare such samples large in size because the sample obtained from one synthesis is very limited. Currently, data of electric resistivity measured by various samples with different thicknesses and sizes is abundant in literature. In fact, the electric resistivity of polyimide is distributed in the range of 10^13^ Ωcm–10^19^ Ωcm [17,18,19,20,21,22]. We consider it important to understand the effect of sample size and thickness on the electric volume resistivity, not only for polyimide, but also other engineering plastics. In addition, understanding the mechanism of electric conduction and the dielectric breakdown behavior of engineering plastics is extremely important in order to improve electrical insulation properties. In this study, we verified some uncertainty regarding the measurement of electric volume resistivity, e.g., film size, thickness, drying time, and the electric field strength, using films of biopolyimide poly(ATA-CBDA) and polyimide (Kapton).

## 2. Experimental Procedures

### 2.1. Synthesis of Biopolyimide

A typical synthetic procedure for biopolyimides, poly(ATA-CBDA), is as follows [14]. 4,4′-diamino-α-truxillic acid dimethyl ester (ATA; 0.20 g, 0.565 mmol) was dissolved in *N,N*-dimethylacetamide (DMAc; 0.56 mL) under a nitrogen atmosphere. Tetracarboxylic dianhydrides such as 1,2,3,4-cyclobutanetetracarboxylic dianhydride (CBDA; 0.11 g, 0.565 mmol) was added into the ATA solution. The reaction mixture was vigorously stirred at room temperature to produce a pale yellow solution and was further stirred for 48 h in order to yield a viscous poly(amic acid) (PAA) solution. The PAA solution was diluted in DMAc and added dropwise into ethanol in order to precipitate PAA fibrils, which were then collected by filtration, thoroughly washed with water, and dried in a vacuum oven for 12 h. For control of the thickness of the polyimide films, a concentration of PAA should be adjusted in this dilution step. The PAA film was obtained by casting a DMAc yellow solution onto a silicon wafer and heating at 60−70 °C. Thermal imidization of the PAA film was carried out by being kept in an oven under a vacuum and stepwise heated at 100, 150, 200, and 250 °C for 1 h at each step. After imidization, a pale yellow poly(ATA-CBDA) film was obtained with a quantitative yield. The detailed information for synthesis of biopolyimides, 1H NMR spectrum, and FT-IR spectrum were shown in Supplementary Materials (Figures S1 and S2).

### 2.2. Electric Resistivity Measurement

**Figure 1:**
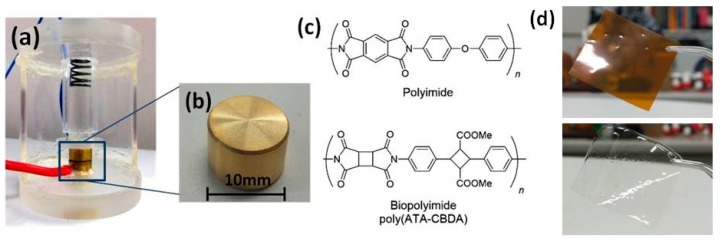
Photographs for (**a**) the laboratory-made cell and (**b**) the electrode for resistivity measurement for polyimide and biopolyimide films with sizes smaller than 40 mm square. (**c**) Chemical structures and (**d**) photographs of polyimide (top) and biopolyimide (bottom) films.

The electric resistivity of polyimide distributed as Kapton^®^ and biopolyimide films was carried out at room temperature using a super megohmmeter (HIOKI DSM-8104) with a resolution of 0.1 fA. A commercial electrode system (HIOKI SME 8311) with an electromagnetic shield was used as an electrode to measure the volume resistance for thickness dependence, electric field dependence, and the drying effect experiment. For the size dependence experiment, a laboratory-made cell with electrodes made of brass (Figure 1) was used to measure the film resistivity for sizes smaller than 40 mm square. A constant electric voltage of 1000 V was applied by the two terminal methods for most experiments, while the electric voltage varied from 100–1000 V for the measurement of electric field dependence. The electric resistivity *ρ*_v_ (Ωcm) was calculated by *ρ*_v_ = (*S*/*t*)*R*_m_, where *S* is the area of the electrode, *t* is the thickness of the film, and *R*_m_ is the measured value of the electric resistance. The time evolution of the electric resistivity of the films was measured. All films were dried in a vacuum oven at 100 °C for at least 3 h before the electric resistivity measurement was conducted.

### 2.3. Dielectric Breakdown Measurement

The dielectric breakdown electric voltage for the poly(ATA-CBDA) films without any drying treatment was measured using an electrical safety analyzer (SE7430 Keisoku Giken) at room temperature. A DC electrical voltage of up to 6 kV was applied along the thickness using the two terminal methods with a ramp-up time of 120 s at a rate of 1 kV s^−1^ using electrodes with a diameter of 20 mm; the maximum current limit was set as 10 mA.

## 3. Results and Discussion

### 3.1. Size Dependency on Electric Resistivity

**Figure 2:**
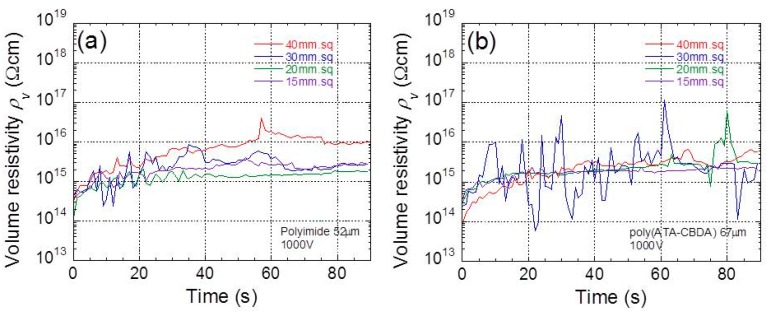
Time profiles of electric resistivity at 1000 V for (**a**) polyimide and (**b**) biopolyimide films with various film sizes.

Figure 2a,b exhibit the time profiles of electric resistivity at 1000 V for polyimide and biopolyimide films with various film sizes, respectively. As seen in the graphs, the electric resistivity showed large noises since the measured current was in the order of 1 pA. The electric resistivity for all films increased over time during the 40 s after the electric voltage was applied. The increase in the resistivity was due to the mobile ions in the film, which is called the charge current, resulting in a macroscopic polarization. At 90 s, only leak current due to the mobile ions occurred in the films, which gives the equilibrium resistivity. No significant decrease in resistivity was observed for films with small sizes. According to our results, the resistivity for synthetic polyimide ranged from 10^15^–10^17^ Ωcm. These values were higher than the values in the literature, which was 10^13^ Ωcm [18], 10^15^~10^16^ Ωcm [19], 10^10^~10^12^ Ωcm [20], or 10^11^ Ωcm [21]. It is natural for the composites of polyimide that, with fillers, the resistivity is largely reduced by conductive ions in the fillers [17,19,21,22]. However, low resistivity is also observed for neat polyimide. It can be considered that there are a few factors involved in the large variation in volume resistivity, e.g., measurement method and drying condition of samples. However, these experimental conditions are not clearly specified in the literature, although it is quite important. We intentionally selected the time profiles of resistivity with comparatively high noise in this paper. The results above show that the resistivity for these films can be measured for small samples, even when the resistivity contains large noises. On the other hand, there is a report showing high resistivity with 5 × 10^19^ Ω cm, with a thickness of 50 μm [17], measured in a vacuum with 10^−6^ Torr. The main reason for the variation of resistivity is probably due to the humidity and the water content in the polyimides.

**Figure 3:**
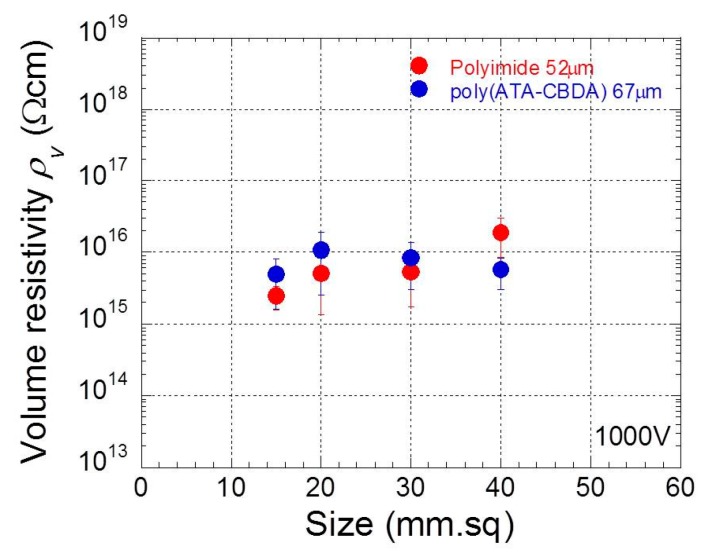
Effect of film size on electric resistivity at 1000 V for polyimide and biopolyimide films.

Figure 3 shows the effect of film size on the average resistivity during 60–90 s in Figure 2 for polyimide and biopolyimide films. The electric resistivity was also averaged over three data sets that were measured by the same film. The electric resistivity for polyimide films with a size of 15 mm square was 2.47 × 10^15^ Ωcm, which was slightly lower than that for the other films. However, the electric resistivity for films larger than 20 mm square was, almost independently of the film size, was approximately 10^16^ Ω cm. This value was one order of magnitude lower than the published value by Dupont [23]. The electric current flowing via the surface (surface conductivity) might slightly affect the volume resistivity for films smaller than 20 mm square. In contrast, it is unneccessary for larger films to pay attention to the surface conductivity. The guidelines by ASTM describe, as a test condition, that the film size should be larger than a square of 80 mm. The result obtained here clearly shows that a small piece of film, 15 mm square, can be used as a sample for the volume resistivity measurement. Biopolyimide films exhibited similar values of resistivity to polyimide, suggesting that the biopolyimide is high insulator as well as polyimide.

### 3.2. Thickness Dependency on Electric Resistivity

**Figure 4:**
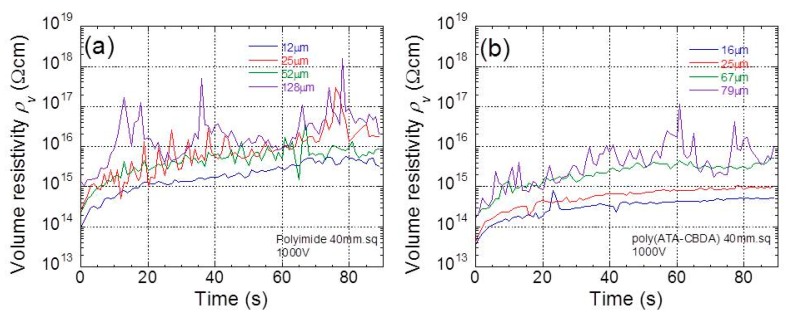
Time profiles of electric resistivity at 1000 V for (**a**) polyimide and (**b**) biopolyimide films with various thicknesses.

Figure 4a,b demonstrate the time profiles of electric resistivity at 1000 V for polyimide and biopolyimide films with various thicknesses, respectively. The sample is a square 40 mm in size. The electric resistivity for all films increased over time during the 40 s after the electric voltage was applied. Similar phenomenon, i.e., charge current and leak current, were also observed in this experiment. It was clearly observed, for both films, that the electric resistivity depends on the film thickness. This strongly indicates that electric resistivity is a function of the electric field strength. 

**Figure 5:**
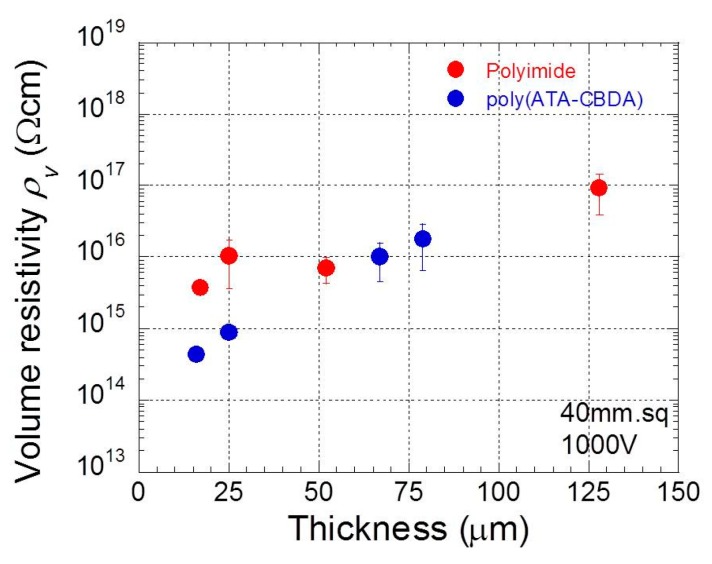
Relation between electric resistivity at 1000 V and thickness for polyimide and biopolyimide films.

Figure 5 indicates the relationship between the averaged resistivity during 60–90 s in Figure 4 and the thickness. The electric resistivity was also averaged over three data sets measured by the same film. The electric resistivity for both polyimide and biopolyimide films decreased remarkably with decreasing thickness, indicating that the mobility of mobile ions was enhanced by the strong Coulomb’s force, due to high electric fields. It was also found, for thin films, that the resistivity for biopolyimide was one order of magnitude lower than that for polyimide.

**Figure 6:**
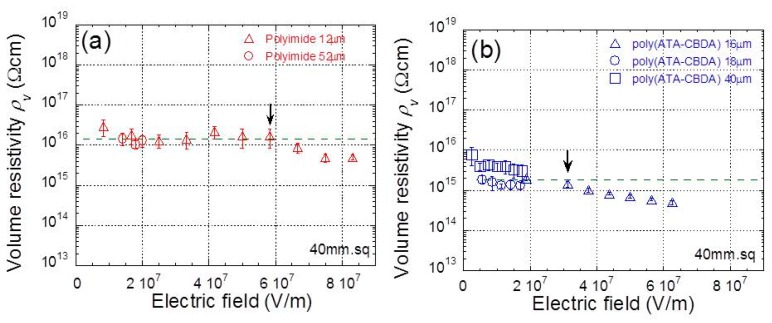
Relationship between electric resistivity and electric field strength for (**a**) polyimide and (**b**) biopolyimide films with different thicknesses.

The electric field strength can be varied by varying the thickness at constant electric voltage or by varying the electric voltage at a constant thickness. Figure 6a,b show the relationship between the averaged electric resistivity and the electric field strength, as a function of the thickness of polyimide and biopolyimide films, respectively. The electric resistivity was averaged over three data sets measured by the same film. Both polyimide and biopolyimide films demonstrated high and constant resistivity at low electric fields and exhibited a gradual decrease at high electric fields. The electric resistivity at low electric fields for biopolyimide was approximately 1/9 lower than that for polyimide. The critical electric field for polyimide and biopolyimide was 5.8 × 10^7^ V/m and 3.2 × 10^7^ V/m, respectively, which suggested that the mobile ions in biopolyimide are easier to move in the film in comparison to that in polyimide. No clear change was found in the IR spectra for biopolyimide films before or after irradiation from a high electric field.

### 3.3. Effect of Drying Time on Electric Resistivity

**Figure 7:**
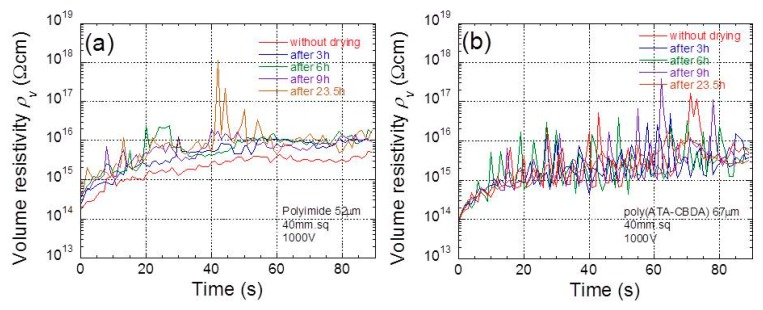
Time profiles of electric resistivity at 1000 V for (**a**) polyimide and (**b**) biopolyimide films with various drying times (film size: 40 mm × 40 mm).

Figure 7a,b exhibit the time profiles of electric resistivity at 1000 V for polyimide and biopolyimide films, respectively, as a function of drying time. Similar to the time profiles of the electric resistivity shown in Figure 2 and Figure 4, the electric resistivity for all films increased over time during the 40 s after the electric voltage was applied, and it was almost constant at 90 s. It is clear that, for both films, the electric resistivity increased with the drying time. It was also found that the amplitude of the noise was independent of the drying time, indicating that the electrical noise was probably caused by the absorbed water in the films. 

**Figure 8:**
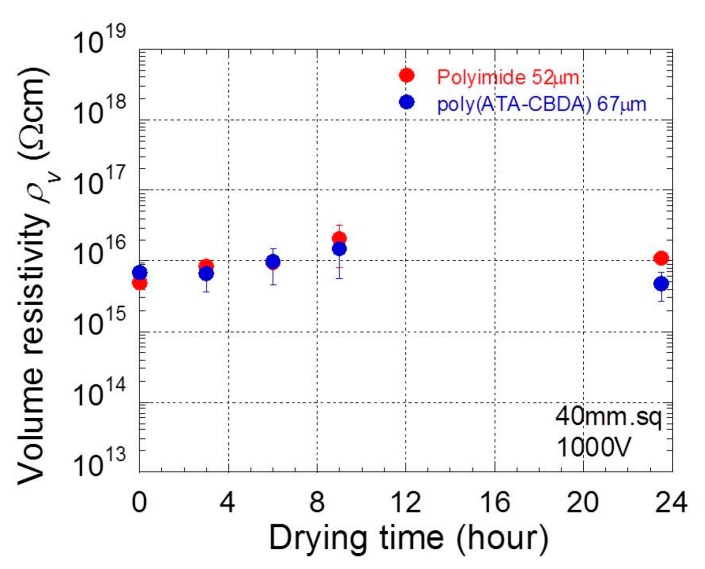
Relationship between electric resistivity at 1000 V and drying time for polyimide and biopolyimide films.

Figure 8 displays the relation between the averaged resistivity at 1000 V and the drying time for polyimide and biopolyimide films. The electric resistivity was averaged over three data sets measured by the same film. The humidity in the experimental room was 34% RH when the experiment was performed. Both polyimide and biopolyimide films demonstrated low electric resistivity (~10^15^ Ωcm) at short drying times, below three hours, and the resistivity increased to 10^16^ Ωcm at longer drying times. This strongly indicates that polyimide and biopolyimide films stored in humidity of 30%–50% absorbed water, and the absorbed water lowers the electric resistivity. In fact, the electric resistivity for polyimide films without drying was significantly reduced to ~10^13^ Ωcm when the humidity was 60% RH.

The electric breakdown electric voltage for polyimide 12 μm and biopolyimide 15 μm films was 3.8 kV and 4.0 kV, respectively, which corresponded to the critical electric field for the dielectric breakdown of 316 MV/m and 266 MV/m, respectively. It was clear that biopolyimide, obtained from biomass, as well as polyimide films synthesized from fossil resources, demonstrate high electrical insulation. The impact of the film sizes, thicknesses, and drying time on the breakdown voltage will be reported in a subsequent paper.

## 4. Conclusions

The electrical insulation property, volume resistivity, and dielectric breakdown electric field, for poly(ATA-CBDA) films obtained from bioresources, was presented. It was clear that the electric resistivity of poly(ATA-CBDA) films showed high electrical insulation properties as well as synthetic polyimide. The electric resistivity measurement exhibited two futures: That a sample larger than 20 mm square is better to determine the volume resistivity and the electric resistivity decreased with the thickness or with increasing the applied electric voltage due to strong electric field strength. The volume resistivity for polyimide and biopolyimide strongly depended on the drying time, suggesting that the resistivity is dominated by the water content absorbed in the films. As mentioned in the Introduction, the poly(ATA-CBDA) film was more transparent than conventional polyimide. We believe that the biopolyimide film with high insulation properties can be used in electronic devices instead of polyimide, which is synthesized from fossil resources.

## Supplementary Materials

The following are available online at https://www.mdpi.com/2073-4360/11/10/1552/s1. Materials and instrument information for synthesis of biopolyimides; Figure S1. ^1^H NMR spectrum of poly(amic acid) which made from 4,4′-diamino-*α*-truxillic acid dimethyl ester (ATA) and 1,2,3,4-cyclobutanetetracarboxylic dianhydride (CBDA) (400 MHz; solvent, DMF-*d*_7_); Figure S2. FT-IR spectrum of (a) poly(amic acid) and (b) poly(ATA-CBDA), as a biopolyimide.
