# Supplementary material for: Electric Volume Resistivity for Biopolyimide Using 4,4′-Diamino-α-truxillic acid and 1,2,3,4-Cyclobutanetetracarboxylic dianhydride
Source: Polymers (Basel). 2019 Sep 24;11(10):1552. doi: 10.3390/polym11101552 (PMC6835271; doi:10.3390/polym11101552)
Supplement: Supplementary file 1 [file polymers-11-01552-s001.pdf]

Supplementary Materials

Electric Volume Resistivity for Biopolyimide Using 4,4'-Diamino-α-truxillic Acid and 1,2,3,4-Cyclobutanetetracarboxylic Dianhydride

Shunsuke KATO [1,†], Fitri Adila AMAT YUSOF [1,†], Toyohiro HARIMOTO [2], Kenji TAKADA [2,†], Tatsuo KANEKO [2,†], Mika KAWAI [1,†] and Tetsu MITSUMATA [1,†,]*

1 Graduate School of Science and Technology, Niigata University, Niigata 950-2181, Japan

2 Graduate School of Advanced Science and Technology, JAIST, Nomi 923-1292, Japan

† ALCA, Japan Science and Technology Agency, Tokyo 102-0076, Japan

* Correspondence: tetsu@eng.niigata-u.ac.jp; Tel.: +81 (0)25 262 6884

CONTENTS

- Materials and instrument information for synthesis of biopolyimides

- ^{1}H NMR spectrum of poly(amic acid) which made from 4,4'-diamino-α-truxillic acid dimethyl ester and 1,2,3,4-cyclobutanetetracarboxylic dianhydride ・・・・・・・・・Figure S1

- FT-IR spectrum of poly(amic acid) and poly(ATA-CBDA) ・・・・・・・・・Figure S2

Materials.

4-Aminocinnamic acid (4ACA) and 1,2,3,4-cyclobutanetetracarboxylic dianhydride (CBDA) were purchased from Tokyo Chemical Industry Co., LTD. *N,N*-Dimethylacetamide were purchased from FUJIFILM Wako Pure Chemical Corporation. Trimethylsilyl chloride (TMS) was purchased from Sigma-Aldrich Co. LLC. CBDA was sublimated by heating (150°C) in *vacuo*. 4,4'-diamino-*α*-truxillic acid dimethyl ester was synthesized based on previous research. [1]. All other chemicals were directly used as purchased.

Instruments.

Proton (^{1}H) nuclear magnetic resonance (NMR) and carbon-^{13} (^{13}C) NMR measurements were carried out using Bruker Bio-spin AG 400 MHz and 100 MHz, respectively. Fourier Transform Infrared Spectroscopy (FT-IR) measurement were recorded with a Perkin-ElmerSpectrum One spectrometer between 4000 and 600 cm^{-1} using a diamond-attenuated total reflection (ATR) accessory.

Reference.

[1] Suvannasara, P.; Tateyama, S.; Miyasato, A.; Matsumura, K.; Shimoda, T.; Ito, T.; Yamagata, Y.; Fujita, T.; Takaya, N.; Kaneko, T. "Bio-based polyimides from 4-aminocinnamic acid photodimer" *Macromolecules* **2014**, *47*, 1586-1593. DOI: 10.1021/ma402499m

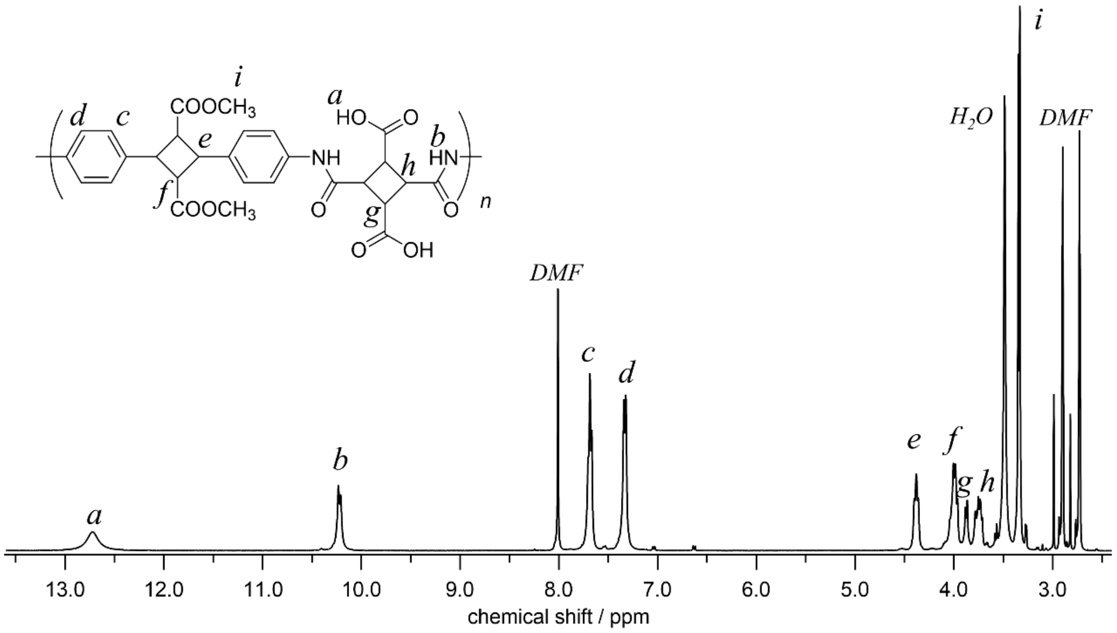

Figure S1. ^{1}H NMR spectrum of poly(amic acid) which made from 4,4'-diamino-α-truxillic acid dimethyl ester (ATA) and 1,2,3,4-cyclobutanetetracarboxylic dianhydride (CBDA) (400 MHz; solvent, DMF-d_7).

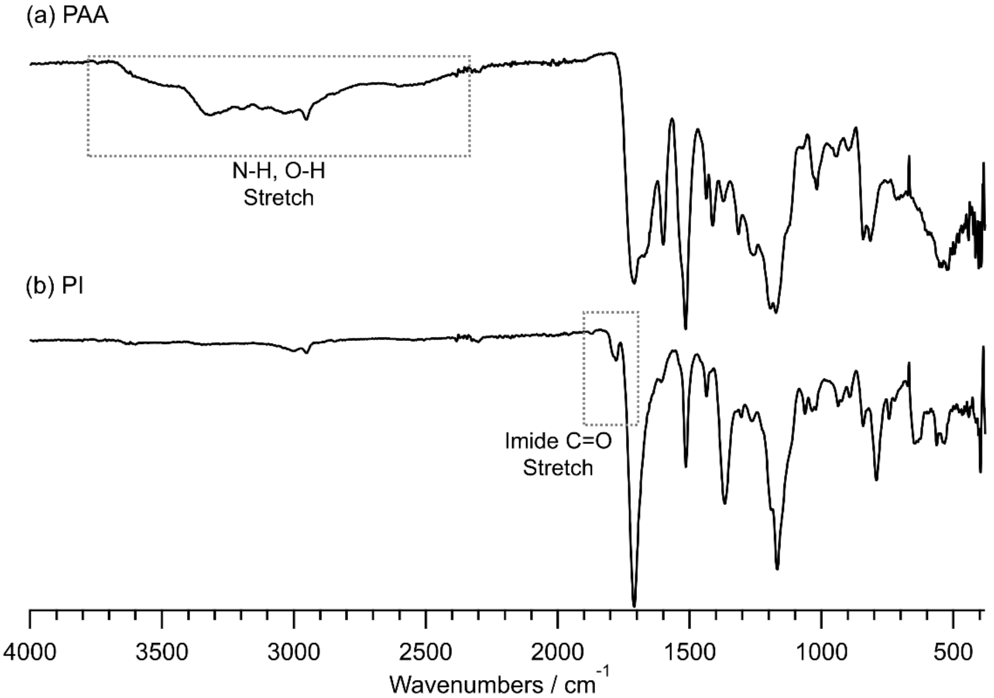

Figure S2. FT-IR spectrum of (a) poly(amic acid) and (b) poly(ATA-CBDA), as a biopolyimide.
